# Impact of selexipag use within 12 months of pulmonary arterial hypertension diagnosis on hospitalizations and medical costs: A retrospective cohort study

**DOI:** 10.1111/crj.13704

**Published:** 2023-10-07

**Authors:** Yuen Tsang, Michael Stokes, Yong‐Jin Kim, Rong Tilney, Sumeet Panjabi

**Affiliations:** ^1^ Real‐World Value & Evidence Janssen Scientific Affairs, LLC Titusville New Jersey USA; ^2^ Data Analytics Evidera St‐Laurent Quebec Canada; ^3^ Data Analytics Evidera Waltham Massachusetts USA

**Keywords:** cost, disease progression, hospitalization, prostacyclin pathway agent, pulmonary arterial hypertension, selexipag

## Abstract

**Background:**

Oral selexipag, a prostacyclin pathway agent (PPA), is effective in patients with pulmonary arterial hypertension (PAH). The objective of this study is to assess the impact of initiating oral selexipag within 12 months of diagnosis on health outcomes.

**Methods:**

This retrospective cohort study used data from Optum's de‐identified Clinformatics® Data Mart Database. PAH patients between 1 October 2015 and 30 September 2019 were included. Patients were also required to have received PAH medication within 12 months of their initial diagnosis. Study groups included patients who initiated selexipag within 12 months of PAH diagnosis (SEL ≤ 12) and those who did not initiate any PPA within 12 months of PAH diagnosis (No PPA ≤ 12). Inverse probability of treatment weighting was used to remove potential confounding between groups. Cox and Poisson regression models were used to compare hospitalization and disease progression. Generalized linear model with gamma distribution and log link was used to compare costs.

**Results:**

SEL ≤ 12 had lower rate of all‐cause hospitalizations (rate ratio: 0.76, 95% confidence interval [CI]: 0.60, 0.96) versus no PPA ≤ 12, but no differences in PAH‐related hospitalization rate (rate ratio: 1.03, 95% CI: 0.79, 1.33) or risk of disease progression (hazard ratio: 1.01, 95% CI: 0.71, 1.44). SEL ≤ 12 incurred lower all‐cause (mean difference: −$23 623; 95% CI: −35 537, −8512) and PAH‐related total medical costs (mean difference: −$12 927; 95% CI: −19 559, −5679) versus no PPA ≤ 12.

**Conclusion:**

Selexipag initiation within 12 months of PAH diagnosis demonstrated reductions in all‐cause hospitalization rate and medical costs.

## Graphical abstract

Prostacyclin pathway agents are effective in improving health outcomes in pulmonary arterial hypertension patients. Availability of oral prostacyclin pathway agents such as selexipag have the potential to benefit patients with less advanced disease. We sought to assess the impact of early selexipag use on hospitalization rates and medical costs in real‐world clinical practice.

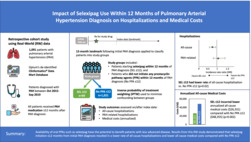

## 1 INTRODUCTION

Pulmonary arterial hypertension (PAH) results in elevated pulmonary vascular resistance and increased pressure in the pulmonary arteries, eventually leading to right ventricular failure and death.^1^, ^2^ Symptoms of PAH include dyspnea, fatigue, chest pain, and syncope.^3^ PAH is a rare disease with approximately 500 to 1000 new cases diagnosed each year in the United States.^4^ Patients diagnosed with PAH have a poor prognosis with 5‐year survival rates of approximately 61%.^5^, ^6^

There is currently no cure for PAH; the treatment goal is to achieve low risk status for expected 1‐year mortality.^2^ Currently available therapies indicated for PAH include prostacyclin pathway agents (PPAs), endothelin receptor antagonists (ERAs), phosphodiesterase‐5 inhibitors (PDE‐5Is), and soluble guanylate cyclase stimulators (sGCs). Current treatment guidelines recommend initial combination therapy with at least two agents approved for PAH.^7^

Research has shown that agents acting on the prostacyclin pathway are effective in improving health outcomes^8^, ^9^, ^10^ but often are underused and initiated too late in the course of the disease history due, in part, to their requirement for continuous parenteral administration and infrequent follow‐up to assess disease progression.^11^ Parenteral PPAs have historically been reserved for severe cases of PAH.^12^, ^13^, ^14^, ^15^ However, availability of oral PPAs introduces the potential to benefit patients in less advanced disease.^12^, ^13^, ^14^ A post hoc analysis of the GRIPHON trial^15^ found that patients who initiated oral selexipag, a PPA that selectively acts on the prostacyclin Ireceptor, within 6 months of PAH diagnosis had a more pronounced treatment effect in delaying disease progression compared with patients who initiated selexipag more than 6 months from diagnosis.^15^ The 2022 European Society of Cardiology and the European Respiratory Society recommended adding oral selexipag in PAH patients with intermediate‐low risk of death at follow‐up while receiving both ERA and PDE5I therapy.^16^

There are limited data assessing the impact of early selexipag use on all‐cause and PAH‐related hospitalization, disease progression, and medical cost outcomes in real‐world clinical practice. The purpose of this study was to address this issue using Optum's Clinformatics® database.

## 2 MATERIALS AND METHODS

### 2.1 Data source

This retrospective cohort study obtained data from Optum's de‐identified Clinformatics® Data Mart Database (OptumInsight, Eden Prairie, MN), an integrated source of medical and prescription records from 180+ million individuals enrolled in UnitedHealth Group private insurance plans. Information is available for enrollees across the United States with data collected from more than 700 hospitals and 7000 clinics. The data are compliant with the Health Insurance Portability and Accountability Act. Therefore, institutional review board approval was not required. Data from 1 April 2015 through 30 September 2020 were retrieved for this analysis.

### 2.2 Study sample

Patients with PAH were identified using a prior algorithm requiring a diagnosis code for pulmonary hypertension (PH) (Table S1) plus a prescription for selexipag or another treatment indicated for PAH (Table S2).^17^ Patients with ≥1 inpatient or ≥2 outpatient claims with an International Classification of Diseases, Ninth Revision (ICD‐9) or International Classification of Diseases, Tenth Revision (ICD‐10) diagnosis code (any position; Table S1) for PH between 1 October 2015 and 30 September 2019 were identified and selected for inclusion. The “initial PAH diagnosis date” was the date of the earliest qualifying PH claim. A 12‐month landmark following the initial PAH diagnosis date was applied to classify patients into the treatment groups. Patients initiating oral selexipag either as monotherapy or combination therapy with other PAH medications on or before the landmark comprised the selexipag use within 12 months (SEL ≤ 12) cohort. Patients who did not initiate any PPA on or before the landmark were included in the comparator cohort (No PPA ≤ 12) and those treated with any PPA other than selexipag were excluded from the No PPA ≤ 12 group. Table S2 lists the PAH‐specific treatments used to classify patients. The landmark time was designated as the study index date for both cohorts.

Patients were excluded if they were not continuously enrolled in their health plan during the pretreatment period, which started 6 months prior to the initial PAH diagnosis date and ended on the landmark time; had evidence of PAH (defined as a relevant diagnosis or receipt of a medication used for PAH) in the 6‐month washout period; had evidence of chronic thromboembolic PH or received any PPAs, lung transplantation, or balloon atrial septostomy during the pretreatment period; were less than 18 years old as of the initial PAH diagnosis; or had a recorded date of death that preceded the landmark time. Figure S1 displays the study timeline and relevant periods used for patient selection.

### 2.3 Outcomes

All outcomes were assessed during the follow‐up period after index. An intention‐to‐treat approach was used for the analysis. Follow‐up began on the index date and continued until the earliest of death, disenrollment, diagnosis of chronic thromboembolic PH, or end of study period. The primary outcome was all‐cause hospitalization.

Secondary outcomes included PAH‐related hospitalization, disease progression, and total allowable medical charges (medical costs). PAH‐related hospitalizations were identified using ICD‐9/10 diagnosis codes (any position) for PH or right heart failure appearing on either the summary hospital or detailed medical claims related to the hospital stay (Table S1). Disease progression was defined as a composite of all‐cause death, PAH‐related emergency room visits/hospitalizations, receipt of parenteral PPA, lung transplantation, or balloon atrial septostomy (Table S3). For patients who met multiple criteria for this outcome, the earliest date on which the composite measure was satisfied was assumed equivalent to the date the measure occurred. All‐cause and PAH‐related total allowable medical charges (standardized “cost”) were defined as the sum of allowable charges associated with claims for any‐cause and PAH‐related medical care (outpatient, inpatient, and emergency department [ED] visits [excluding pharmacy costs]). Costs were analyzed on a per patient per year basis and reported in 2019 US dollars. Claims related to medical care for PAH were identified using ICD‐9/10 diagnosis codes (any position).

### 2.4 Statistical analysis

Inverse probability of treatment weighting (IPTW) was used to address the imbalance of potential confounding factors between the SEL ≤ 12 and No PPA ≤ 12 groups. IPTW removes confounding by creating a “pseudo‐population” (i.e., weighted population) in which the treatment is independent of the measured confounders. Propensity scores (PS) representing the probability of assignment into the SEL ≤ 12 group were estimated using a multivariable logistic regression model. Baseline characteristics including age, gender, comorbidities, index diagnosis year, Charlson comorbidity index (CCI),^18^ PAH medication use, healthcare resource use, and allowable charges were included in the model.^19^ Baseline characteristics were assessed during the 12‐month period prior to the study index date. For each individual patient, stabilized weights equal to the inverse of their PS were calculated. Comparisons of the baseline clinical and demographical characteristics were conducted before and after applying IPTW using standardized differences. A standardized difference of less than 0.20 was used to indicate good balance.

Each study outcome was compared between the treatment groups. Statistics for all outcome measures were weighted using IPTW. Rates of all‐cause and PAH‐related hospitalizations were expressed as total number of hospitalizations per 100 person‐years. Calculations of rates included the initial and any subsequent clinical events. All‐cause and PAH‐related hospitalization rates were modeled using multivariable Poisson regression, with follow‐up time as an offset variable to derive rate ratios with corresponding 95% confidence intervals (CIs) and *p*‐values. Kaplan–Meier estimates of survival probabilities were plotted to examine the time to all‐cause and PAH‐related hospitalizations as well as disease progression and compared using log‐rank tests. Hazard ratios (HR) with 95% CIs for the risk of each clinical event were estimated using multivariable Cox proportional hazards regression.

Costs were analyzed on a per patient per year basis to account for differential follow‐up. Costs were reported in 2019 US dollars; inflation adjustments were made using the medical component of the Consumer Price Index.^20^ A multivariable generalized linear model with Gamma distribution and log link function was used to estimate the mean cost differences for hospitalization (all‐cause and PAH‐related) and total medical costs (all‐cause and PAH‐related) between the cohorts, along with their 95% CIs.

The multivariable regression models were adjusted to account for differences in potential confounding characteristics between cohorts that persisted following IPTW. Adjustment for multiple comparisons was not conducted. The following baseline parameters were included in each model: year of diagnosis, idiopathic PAH and/or heritable PAH, left ventricular dysfunction, other pulmonary diseases with mixed restrictive and obstructive pattern, Raynaud's syndrome, liver disease, total length of stay (days), duration between initial PAH diagnosis and initiation of treatment, total PAH medication days supplied, and total allowable medical charges. Statistical significance of differences was evaluated at the alpha = 0.05 level. All analyses were conducted using SAS V9.4 or higher statistical software (SAS Institute, Cary, NC).

## 3 RESULTS

**FIGURE 1:**
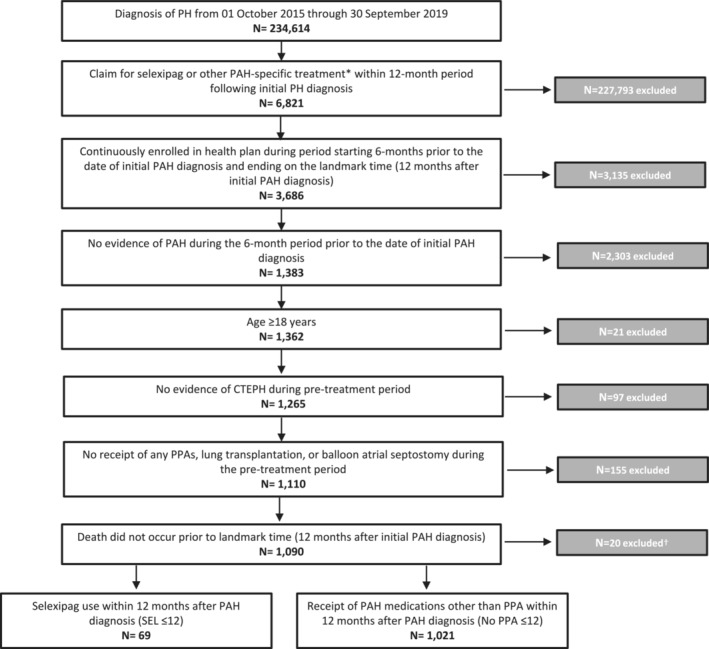
Sample attrition. *Including other PPAs, endothelin receptor agonists, phosphodiesterase‐5 inhibitors, or soluble guanylate cyclase stimulators. ^†^Patients excluded were those that had a recorded date of death prior to landmark time and also had complete enrollment during the landmark period. It is not uncommon for health plans to terminate coverage on the last day of the month of the member's death. Hence, it is possible for some patients to be enrolled for a period of time after their recorded date of death. CTEPH, chronic thromboembolic pulmonary hypertension; PAH, pulmonary arterial hypertension; PH, pulmonary hypertension; PPA, prostacyclin pathway agent.

From an initial sample of 6821 patients with evidence of PAH from 1 October 2015 through 30 September 2019, 1090 patients fulfilled the eligibility criteria and were included in the study (Figure 1). Of these, 69 and 1021 patients were classified as SEL ≤ 12 and No PPA ≤ 12 cohorts, respectively.

Before IPTW adjustment, patients in the SEL ≤ 12 group were, on average, slightly younger (mean [standard deviation {*SD*}] age: 67.6 [12.4] years vs. 69.8 [12.1] years, *p* = 0.141), more likely to be female (69.6% vs. 62.5%, *p* = 0.2388), and had a comparable mean (*SD*) CCI score (7.2 [3.6] vs. 7.0 [3.6], *p* = 0.7393) relative to those in No PPA ≤ 12 group (Table 1). Patients in SEL ≤ 12 group also had a higher mean (*SD*) number of baseline all‐cause hospitalizations (2.7 [1.9] vs. 2.2 [1.6], *p* = 0.1050) and PAH‐related hospitalizations (0.4 [0.8] vs. 0.2 [0.4], *p* = 0.0235) as well as higher use of two or more PAH medication classes (2: 40.6% vs. 18.4%, *p* < 0.0001; 3: 40.6% vs. 0.5%, *p* < 0.0001; 4+: 1.4% vs. 0%, *p* = 0.0633) compared with those in No PPA ≤ 12 group. The most commonly used therapies in SEL ≤ 12 were combination therapies and included selexipag + ERA + PDE‐5I (33.3%), selexipag + PDE‐5I (20.3%), and selexipag + ERA (17.4%). The majority of patients in the No PPA ≤ 12 group received treatment with only one medication class (ERA 11.8%, sGC 4.5%, or PDE‐5I 64.8%). The most common combination therapy was ERA + PDE‐5I (14.7%) and ERA + sGC (3.1%) (Table 1). Patients in No PPA ≤ 12 group had significantly longer mean (*SD*) duration between initial PAH diagnosis and initiation of PAH treatment relative to those in SEL ≤ 12 group (118.0 [103.6] days vs. 55.0 [59.0] days, *p* < 0.0001). The majority of patients included in the study resided in either the South (49.8%) or West (25.4%) regions and were enrolled in Medicare (71%).

**TABLE 1:** Baseline demographic and clinical characteristics before and after inverse probability of treatment weighting.

Characteristics (%)	Unweighted				Weighted			
	SEL ≤ 12	No PPA ≤ 12	Standardized difference	*p* value	SEL ≤ 12	No PPA ≤ 12	Standardized difference	*p* value
Number of individuals	69	1021			70	1013		
Mean age, years (*SD*)	67.6 (12.42)	69.8 (12.08)	0.181	0.141	71.1 (11.31)	69.7 (12.09)	−0.1228	0.3357
Median (IQR)	70 (60–76)	71 (63–79)			74 (65–78)	71 (62–79)		
Male	30.4%	37.5%	0.1499	0.2388	33.7%	37.0%	0.0696	0.5781
Geographic region								
Northeast	4.3%	8.1%	0.1568	0.3565	3.2%	8.2%	0.2175	0.1334
North Central	20.3%	16.6%	−0.0965	0.4215	19.4%	16.7%	−0.0712	0.5554
South	55.1%	49.5%	−0.1125	0.3669	64.2%	49.4%	−0.3027	0.0167
West	18.8%	25.9%	0.169	0.1951	12.6%	25.7%	0.3369	0.0146
Unknown/missing	1.4%	0.0%	−0.1715	0.0633	0.5%	0.0%	−0.1025	0.0213
Payer type								
Commercial	29%	21.8%	−0.1646	0.1676	23.2%	22%	−0.3371	0.0007
Medicare	71%	78.2%	0.1646	0.1676	76.8%	78%	0.3371	0.0007
Mean CCI score (*SD*)	7.2 (3.60)	7.0 (3.62)	−0.0415	0.7393	7.4 (3.42)	7.0 (3.59)	−0.1063	0.4011
Comorbidities								
Portal hypertension	2.9%	3.6%	0.0408	1.0000	4.3%	3.6%	−0.0351	0.7686
Congenital heart disease	14.5%	11.0%	−0.1058	0.3690	14.0%	10.9%	−0.0968	0.4112
COPD	47.8%	47.1%	−0.0143	0.9083	59.1%	47.2%	−0.239	0.0558
ILD	14.5%	14.2%	−0.0083	0.9466	19.6%	14.2%	−0.1444	0.2167
Diabetes	39.1%	50.4%	0.2289	0.0690	45.4%	49.9%	0.0896	0.4707
Obesity	33.3%	46.3%	0.2678	0.0359	39.2%	45.4%	0.1261	0.313
Hypertension	92.8%	91.0%	−0.0646	0.8268	95.3%	91.0%	−0.1683	0.2251
Mean duration between initial diagnosis and initiation of PAH treatments (days)	55.0 (59.0)	118.0 (103.6)	0.7479	<0.0001	88.8 (70.2)	114.9 (102.4)	0.2979	0.0047
PAH medication								
ERA	59.4%	30.1%	−0.6178	<0.0001	53.5%	31.9%	−0.4493	0.0002
PDE5I	55.1%	80.6%	0.5683	<0.0001	57.9%	80.9%	0.5146	<0.0001
Riociguat	11.6%	8.7%	−0.0954	0.4166	7.5%	9.2%	0.0614	0.6338
PAH treatment regimen								
Monotherapy	17.4%	81.1%	1.6533	<0.0001	86.0%	77.6%	−0.2209	0.0537
Doublet therapy	40.6%	18.4%	−0.5011	<0.0001	11.4%	20.6%	0.2516	0.031
Triplet therapy	40.6%	0.5%	−1.1431	<0.0001	2.4%	1.8%	−0.043	0.6678
Quadruplet or higher therapy	1.4%	0.0%	−0.1715	0.0633	0.1%	0.0%	−0.0364	0.4137
Mean PAH medication days supplied, days (*SD*)	311.1 (230.13)	217.7 (165.35)	−0.4662	0.0014	289.9 (171.75)	225.1 (170.31)	−0.3784	0.0022
Mean number of all‐cause hospitalizations (*SD*)	2.7 (1.9)	2.2 (1.6)	−0.1422	0.1050	1.1 (1.4)	1.1 (1.6)	0.017	0.8957
Mean total allowable medical charges in USD (*SD*)	69 836.6 (79 957.81)	80 289.0 (116 344.2)	0.1047	0.3126	51 570.2 (54 045.93)	80 453.9 (116 727.0)	0.3176	0.0002

Abbreviations: CCI, Charlson comorbidity index; COPD, chronic obstructive pulmonary disease; ERA, Endothelin receptor antagonists; ILD, interstitial lung disease; PAH, pulmonary arterial hypertension; PDE5i, Phosphodiesterase‐5 inhibitors; PPA, prostacyclin pathway agent; SD, standard deviation; IQR, interquartile range; USD, US dollars.

After IPTW, both cohorts were balanced on most of the demographic and clinical characteristics that were included in the PS model. These included year of diagnosis, age, sex, portal hypertension, congenital heart diseases, chronic obstructive pulmonary disease, interstitial lung disease, diabetes, obesity, hypertension, number of all‐cause hospitalizations, duration between initial diagnosis and initiation of PAH treatment, and number of additional PAH medication classes received. After IPTW, the median follow‐up was 18 months for SEL ≤ 12 and 14 months for No PPA ≤ 12 groups.

### 3.1 Hospitalization rate and risk (weighted study population)

**FIGURE 2:**
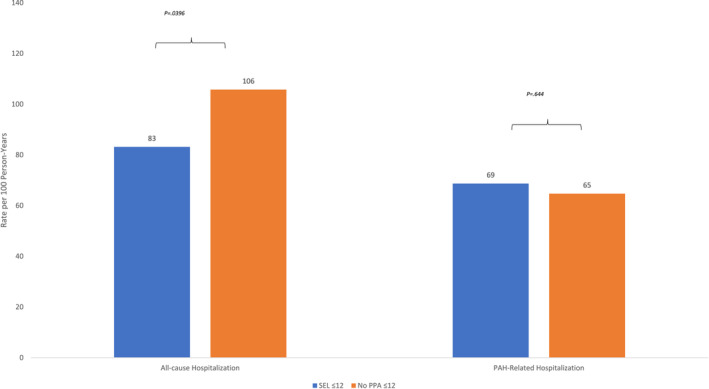
Rate of hospitalizations (weighted study population). PAH, pulmonary arterial hypertension; PPA, prostacyclin pathway agent.

The cumulative all‐cause hospitalization rates were 83.2 and 105.8 per 100 person‐years for SEL ≤ 12 and No PPA ≤ 12 groups, respectively (*p* = 0.0396) (Figure 2). PAH‐related hospitalization rates were 68.7 and 64.7 per 100 person‐years for SEL ≤ 12 and No PPA ≤ 12 groups, respectively (*p* = 0.644).

**FIGURE 3:**
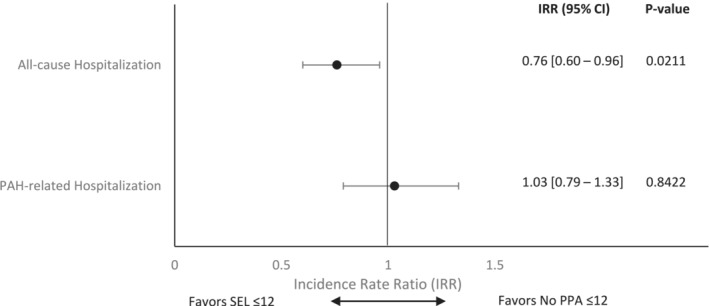
Adjusted multivariable models examining the incidence rate ratio for hospitalizations (all‐cause and PAH‐related) with SEL ≤ 12 versus no PPA ≤ 12 (weighted study population). PAH‐related hospitalizations were identified using PAH diagnosis codes appearing on both the summary hospital and individual medical claims submitted during patients' inpatient stay. The multivariable regression models included year of diagnosis, idiopathic PAH and/or heritable PAH, left ventricular dysfunction, other pulmonary diseases with mixed restrictive and obstructive pattern, Raynaud's syndrome, liver disease, total length of stay (days), duration between initial PAH diagnosis and initiation of treatment, total PAH medication days supplied, and total allowable medical charges to account for potential confounding characteristics that persisted following inverse probability of treatment weighting. PAH, pulmonary arterial hypertension; PPA, prostacyclin pathway agent.

After adjusting for potential confounders that remained imbalanced following IPTW, patients in SEL ≤ 12 cohort had a 24% lower rate of all‐cause hospitalizations compared with the No PPA ≤ 12 cohort (incidence rate ratio [IRR] 0.76, 95% CI: 0.60, 0.96, *p* = 0.021) (Figure 3). The rate of PAH‐related hospitalizations was similar between the study cohorts (IRR 1.03, 95% CI: 0.79, 1.33, *p* = 0.8422).

**FIGURE 4:**
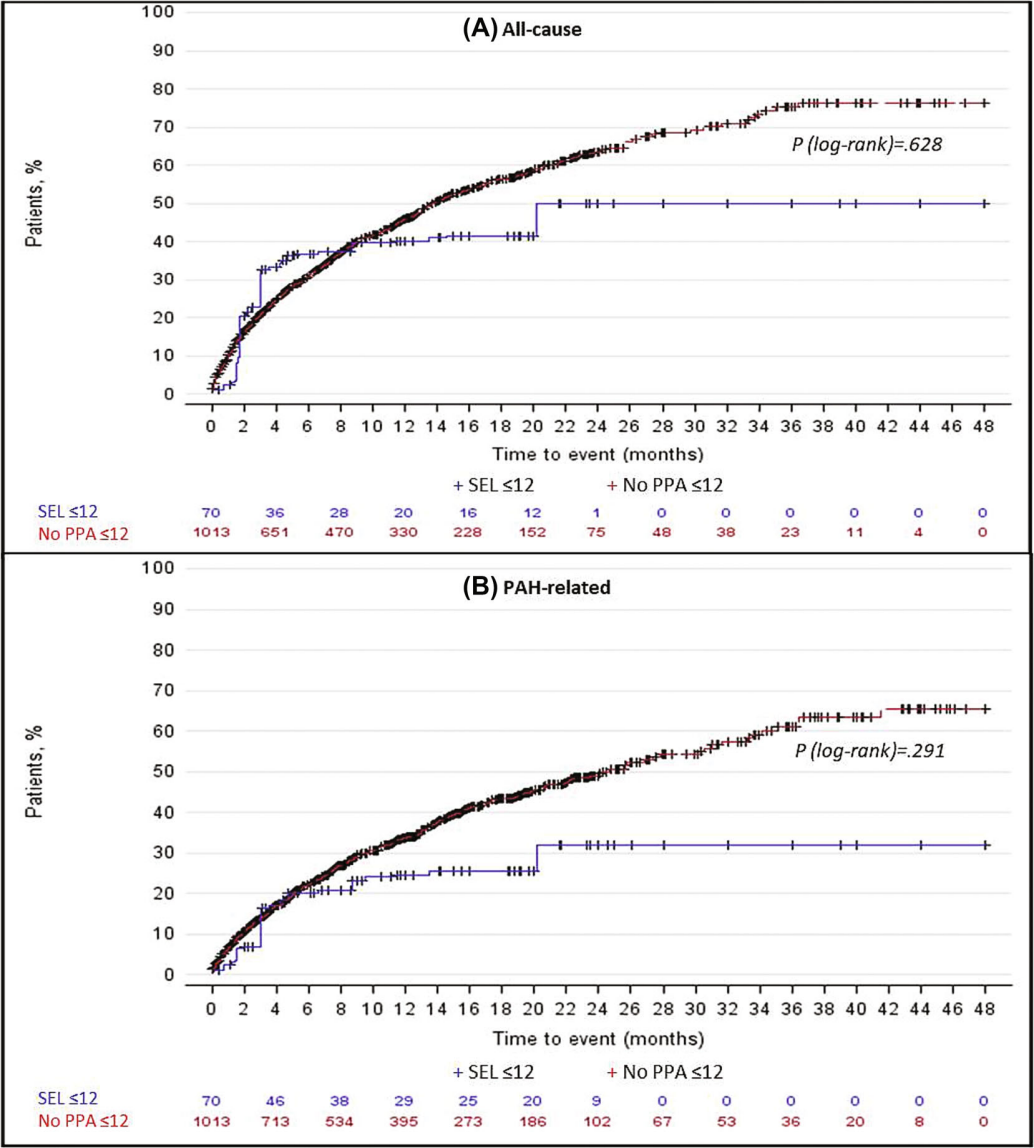
Kaplan–Meier analysis of time to hospitalization (weighted study population). (A) All‐cause. (B) PAH‐related. PAH‐related hospitalizations were identified using PAH diagnosis codes appearing on both the summary hospital and individual medical claims submitted during patients' inpatient stay. PAH, pulmonary arterial hypertension; PPA, prostacyclin pathway agent.

**FIGURE 5:**
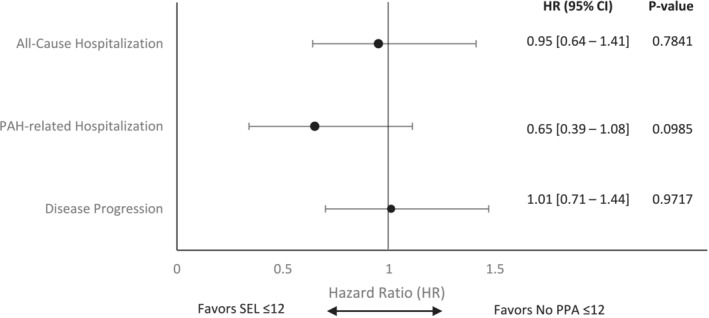
Adjusted multivariable models examining the hazard ratio for hospitalizations (all‐cause and PAH‐related) and disease progression with SEL ≤ 12 versus no PPA ≤ 12 (weighted study population). PAH‐related hospitalizations were identified using PAH diagnosis codes appearing on both the summary hospital and individual medical claims submitted during patients' inpatient stay. The multivariable regression models included year of diagnosis, idiopathic PAH and/or heritable PAH, left ventricular dysfunction, other pulmonary diseases with mixed restrictive and obstructive pattern, Raynaud's syndrome, liver disease, total length of stay (days), duration between initial PAH diagnosis and initiation of treatment, total PAH medication days supplied, and total allowable medical charges to account for potential confounding characteristics that persisted following inverse probability of treatment weighting. CI, confidence interval; PAH, pulmonary arterial hypertension; PPA, prostacyclin pathway agent.

The median time to all‐cause hospitalization was not reached for patients in SEL ≤ 12 group, while it was 13.8 months (95% CI: 12.4–16.0) for No PPA ≤ 12 group (Figure 4A). At 48 months, the cumulative probability of hospitalization for any cause was 50% for SEL ≤ 12 and 76.4% for No PPA ≤ 12. After adjusting for potential confounders that remained imbalanced following IPTW, the hazard of all‐cause hospitalization (HR 0.95, 95% CI: 0.64, 1.41, *p* = 0.784) between the study cohorts was not statistically significant (Figure 5). The median time to PAH‐related hospitalization was not reached for SEL ≤ 12 group, and it was 24.7 months (95% CI: 20.7–30.5) for the No PPA ≤ 12 group (Figure 4B). The cumulative probability of a PAH‐related hospitalization was 32% for SEL ≤ 12 and 65.6% for No PPA ≤ 12 group at 48 months. A lower hazard of PAH‐related hospitalization (HR 0.65, 95% CI: 0.39, 1.08) was observed for patients in No PPA ≤ 12 compared with SEL ≤ 12 cohort (Figure 5). However, the result was not statistically significant (*p* = 0.099).

### 3.2 Disease progression (weighted study population)

**FIGURE 6:**
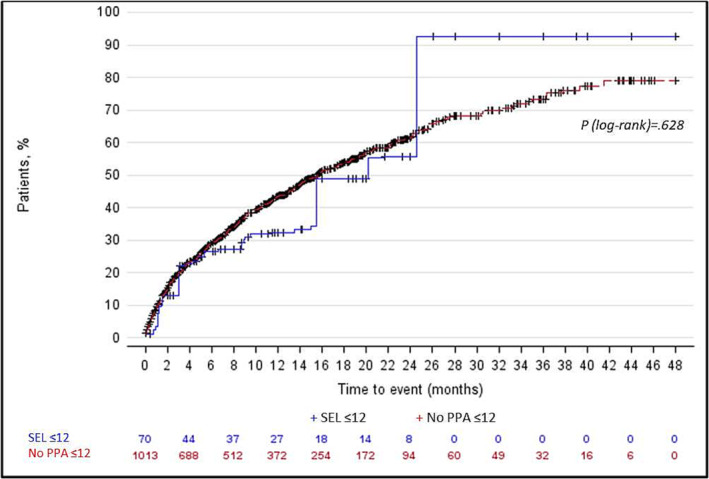
Kaplan–Meier analysis of time to disease progression (weighted study population). Disease progression included all‐cause death, PAH‐related hospitalization, PAH‐related emergency department visits, receipt of parenteral PPA, lung transplantation, and/or balloon atrial septostomy. PAH‐related hospitalizations were identified using PAH diagnosis codes appearing on both the summary hospital and individual medical claims submitted during patients' inpatient stay. PAH, pulmonary arterial hypertension; PPA, prostacyclin pathway agent.

Median time to disease progression was longer for patients in SEL ≤ 12 (20.2 months, 95% CI: 5.30–nonestimable) compared with those in No PPA ≤ 12 (15.6 months, 95% CI: 13.9–18.0), although the results were not statistically significant (*p* = 0.968) (Figure 6). The cumulative probability of disease progression was 92.6% for SEL ≤ 12 and 79% for No PPA ≤ 12 at 48 months. There was no difference in the hazard of disease progression (HR 1.01, 95% CI: 0.71, 1.44, *p* = 0.917) between the study cohorts after adjusting for potential confounders (Figure 5).

### 3.3 Total medical costs (weighted study population)

**FIGURE 7:**
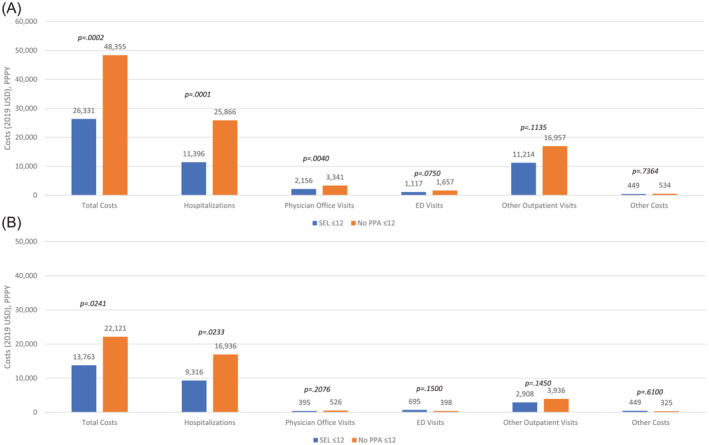
Medical costs among SEL ≤ 12 and No PPA ≤ 12 groups (weighted population). (A) All‐cause. Costs for skilled nursing facility and long‐term care were included in the “other costs” category. (B) PAH‐related. Costs for skilled nursing facility and long‐term care were included in the “other costs” category. PAH‐related hospitalizations were identified using PAH diagnosis codes appearing on both the summary hospital and individual medical claims submitted during patients' inpatient stay. The multivariable generalized linear models included year of diagnosis, idiopathic PAH and/or heritable PAH, left ventricular dysfunction, other pulmonary diseases with mixed restrictive and obstructive pattern, Raynaud's syndrome, liver disease, total length of stay (days), duration between initial PAH diagnosis and initiation of treatment, total PAH medication days supplied, and total allowable medical charges to account for potential confounding characteristics that persisted following inverse probability of treatment weighting. ED, emergency department; PAH, pulmonary arterial hypertension; PPA, prostacyclin pathway agent; PPPY, per person per year.

Overall, the SEL ≤ 12 cohort exhibited lower annualized all‐cause total medical costs ($26 331) compared with No PPA ≤ 12 ($48 355) (Figure 7A). Patients in SEL ≤ 12 cohort had lower costs across all‐cause healthcare utilization categories relative to No PPA ≤ 12 (hospitalizations: $11 396 vs. $25 866 [*p* = 0.0001]; physician office visits: $2156 vs. $3341 [*p* = 0.0040]; ED visits: $1117 vs. $1657 [*p* = 0.0750]; other outpatient visits: $11 214 vs. $16 957 [*p* = 0.1135]; other costs: $449 vs. $534 [*p* = 0.7364], respectively). A similar trend was observed for PAH‐related costs, except for ED visits, where the associated costs were higher for the SEL ≤ 12 cohort ($695) compared with No PPA ≤ 12 ($398) (*p* = 0.1500) (Figure 7B). The main drivers of costs included hospitalizations (SEL ≤ 12: 43% and 68%; No PPA ≤ 12: 54% and 77% of total all‐cause and PAH‐related costs, respectively) and other outpatient visits (SEL ≤ 12: 43% and 21%; No PPA ≤ 12: 35% and 18% of total all‐cause and PAH‐related costs, respectively) (Figures 7 and S2).

After adjusting for potential confounding that persisted following IPTW, SEL ≤ 12 incurred $23 623 (95% CI: 8512, 35 537, *p* = 0.006) lower total all‐cause medical costs than No PPA ≤ 12. Similarly, the mean total medical cost difference from the generalized linear model showed that patients in the SEL ≤ 12 cohort incurred $12 927 (95% CI: 5679, 19 559, *p* = 0.006) lower PAH‐related costs than No PPA ≤ 12.

## 4 DISCUSSION

We used data from the Optum Clinformatics® claims database to assess the risk of hospitalization (all‐cause and PAH‐related), disease progression, and total allowable medical charges (exclusive of prescription drugs) between patients initiating selexipag within 12 months of their PAH diagnosis and patients who did not start treatment with any PPAs within 12 months of diagnosis. Increasingly, real‐world evidence is being recognized by healthcare and regulatory decision‐makers as an important source of evidence, complementary to results obtained from randomized clinical trials.

In this analysis, there were no significant differences in all‐cause hospitalization risk (HR 0.95, 95% CI: 0.64, 1.41, *p* = 0.7841), disease progression risk (HR 1.01, 95% CI: 0.71, 1.44, *p* = 0.9717), and PAH‐related hospitalization rate (IRR 1.03, 95% CI: 0.79, 1.33, *p* = 0.8422) between selexipag users and patients who did not receive PPA. PAH‐related hospitalization risk (HR 0.65, 95% CI: 0.39, 1.08, *p* = 0.0985) was reduced with selexipag versus no PPA but did not achieve statistical significance. Statistically significant reduction in all‐cause hospitalization rate (IRR 0.76, 95% CI: 0.60, 0.96, *p* = 0.0211) and total medical costs (see Figure 7) were observed with selexipag versus no PPA. While rate and risk are similar measures, the hospitalization rate captures all events (initial and subsequent) and accounts for observable patient‐time, while only the first event is captured with risk. Patients may be susceptible to multiple all‐cause hospitalization events, some of which may be prevented with selexipag, while the overall risk of all‐cause hospitalization is similar between the treatment groups. In contrast, we observed a trend of reduced PAH‐related hospitalization risk with selexipag. However, the rate of PAH‐related hospitalizations was similar between study groups. This may indicate an increased number of PAH‐related admissions in a subset of selexipag patients, potentially unresponsive to treatment. Only about 19% of patients in the no PPA group received a combination therapy for PAH despite the treatment guidelines recommending initial treatment with combination therapy.

Analyses of baseline characteristics prior to IPTW indicate that physicians may have selectively prescribed selexipag to patients with more severe PAH as measured by healthcare utilization (PAH‐related hospitalizations and number of PAH medication classes received) and comorbidities (CCI). For instance, compared with patients who were not treated with PPA, selexipag users had a higher mean number of all‐cause hospitalizations during baseline, received initial treatment more quickly after PAH diagnosis, and were more likely to have received double and triple combination therapies for PAH (including PPA). However, it is difficult to determine the clinical reasons for the assigned treatment because the indication for treatment based on physician and patient perceptions of disease severity and prognosis are not available in the data.

This study suggests a reduction in all‐cause hospitalization rates and total medical costs among patients who initiate selexipag within 1 year of PAH diagnosis and those who do not. Although the above findings should be interpreted with caution due to the inherent limitations of claims database analyses, this study complements the results from a post hoc analysis of the GRIPHON trial that showed a more pronounced treatment effect on delaying disease progression for patients that initiated selexipag within 6 months from diagnosis (HR: 0.45; 95% CI: 0.33, 0.63) compared with patients that initiated selexipag with a longer time from diagnosis (HR: 0.74; 95% CI: 0.57, 0.96).^15^ Although our study did not find a treatment effect on disease progression, a reduction in the all‐cause hospitalization rate and total medical costs with selexipag was observed. However, in the post hoc pooled analysis of the Phase III GRIPHON^21^ and Phase IIIb TRITON^22^ trials, early use of selexipag was associated with a 52% reduced risk of disease progression compared with placebo (HR 0.48; 95%CI: 0.35, 0.66).^23^ The reasons for the difference between our findings and those of the above post hoc pooled analysis are unclear. One possible explanation, in part, for the difference in treatment effect is the unselected population observed in the claims database and absence of randomization. Patients in real‐world settings are generally older and have more comorbidity compared with those enrolled in clinical trials. Additionally, the definition and components of disease progression that varied between the pooled post hoc analysis and our study also played a role. In our study, the disease progression events were measured with a healthcare administrative claims database that provided an overview of patients' medical diagnosis, procedure, healthcare, and pharmacy utilization histories which is not as rich in clinical detail as clinical trial data (e.g., GRIPHON and TRITON trials). For instance, the disease progression events in both GRIPHON and TRITON trials used 6‐min walk distance and functional class that were adjudicated by a clinical events committee and not available in administrative claims data.^21^, ^22^ Another possible reason for the difference in treatment effect on disease progression between the two studies is the timing of selexipag initiation (i.e., within 6 months of diagnosis for post hoc pooled analysis vs. within 12 months of diagnosis for the present study) and study population (i.e., clinical trial for post hoc pooled analysis vs. real‐world clinical setting for present study). Finally, the current study did not directly compare patients receiving selexipag ≤12 months after PAH diagnosis to those receiving selexipag >12 months. This approach would have resulted in unbalanced treatment groups with respect to time from PAH diagnosis to initiation of selexipag, and potentially skewing results in favor of the SEL ≤ 12 group. Instead, we opted for the more conservative landmark design to ensure outcomes were estimated for all patients starting at the same point in time.

Although our study used IPTW to adjust for variables not sufficiently balanced between the two cohorts, these models are dependent on observed covariates, and thus, any confounders not identifiable in the data were not accounted for. The results may be influenced by unmeasured confounders that have been demonstrated to predict outcomes in PAH such as 6‐min walk distance, World Health Organization functional class, exercise capacity, hemodynamics, and biomarkers.^24^, ^25^

Additionally, claims data are primarily collected for reimbursement purposes rather than research purposes. Information on claims is subject to errors of omission and/or commission. The ICD coding systems only have a code corresponding to PH; they lack the specificity to identify patients with the PAH subtype. To overcome this limitation, we used a previous algorithm that required diagnoses of PH and at least one claim for a medication indicated for PAH.^17^ Because our cohort only comprises patients treated with a PAH‐related medication, we expect misclassification of patients to be minimal as most healthcare payers will require a confirmed PAH diagnosis prior to administration.^26^, ^27^

Although we used a 6‐month washout period to identify incident cases of PAH, without access to patients' entire medical history, it is possible that some patients in the study sample may have been misclassified; it is also possible that the date of diagnosis in the claims does not accurately reflect the date of clinical recognition of PAH. Another limitation of pharmacy claims includes availability of data to indicate that a medication was dispensed, but not whether or how it was used. Healthcare claims also do not include information on medications administered during hospitalizations.

A landmark design was used because the study groups were determined based on treatments received within 12 months following the PAH diagnosis. This method ensures that outcomes are estimated for all patients starting at the same point in time, the 12‐month landmark. However, this design choice also limits generalizability of results as only patients who survive to the 12‐month landmark and remain with the same insurance provider are included in the analysis.

Identification of hospitalizations related to PAH can be challenging using diagnosis codes as patients with PAH usually have multiple comorbidities and it is not always clear whether it was the comorbidity or disease that led to the hospitalization event. It is possible that some PAH hospitalizations were not correctly identified or their costs were influenced by the presence of other conditions. A similar complexity is also observed in the REVEAL study in which hospitalization events were reviewed by three investigators and categorized as PAH related or “PAH unrelated.” Although the investigators in the REVEAL study found that 53% of the PAH patients experienced a PAH‐related hospitalization event, the authors of the study state that it is unclear whether the “PAH‐unrelated” hospitalization was triggered by PAH to some degree.^28^

With regard to limitations specific to the Optum Clinformatics® database, only “standardized cost” is reported which reflects allowable charges rather than the cost of care. The completeness of death data in the Optum Clinformatics® database is not accurate beyond 2013 due to the removal of the mandatory requirement of reporting deaths. This may have impacted adjudication of disease progression events in our study. While large and geographically diverse, the database represents a convenience—as opposed to a randomized—sample, with concerns about bias and generalizability. The sample within the Optum Clinformatics® database consists mainly of commercially insured patients. Our study had a small sample size especially in the SEL ≤ 12 group (69 patients). Therefore, the study results might not be generalizable to the Medicaid and Medicare fee‐for‐service populations, especially for patients ≥65 years old.

The small sample size of patients that initiated selexipag within 12 months of PAH diagnosis raises concerns because of the error associated with the estimation of the all‐cause and PAH‐related hospitalization risk and violation of the proportional hazards assumption. To address this limitation, we conducted a series of analyses that included introducing time‐varying covariates in the Cox proportional hazards models and using accelerated time failure models with various distribution (i.e., exponential, log‐logistic, and Weibull). However, the results of these sensitivity analyses followed the same trend as the main analyses, an encouraging sign that the study methods used were robust.

## 5 CONCLUSION

This study suggests a reduction in all‐cause hospitalization rates and all‐cause and PAH‐related medical costs in patients who received selexipag within 12 months of diagnosis compared with patients who did not receive PPAs. The PAH‐related hospitalization rate was not different between the study groups and did not achieve statistical significance. Only 6.4% of the study sample received selexipag indicating an overall underutilization of PPAs. The finding of reduced medical costs and all‐cause hospitalization rates among patients that initiated selexipag within 12 months of PAH diagnosis may be of interest to healthcare decision‐makers and payers.

## AUTHOR CONTRIBUTIONS

**Yuen Tsang:** Conceptualization; investigation; methodology; project administration; supervision; writing—original draft; writing—review and editing. **Michael Stokes:** Conceptualization; investigation; methodology; supervision; writing—original draft; writing—review and editing. **Yong‐Jin Kim:** Conceptualization; investigation; methodology; supervision; writing—original draft; writing—review and editing. **Rong Tilney:** Formal analysis; methodology; software; validation. **Sumeet Panjabi:** Conceptualization; investigation; methodology; supervision; writing—review and editing.

## CONFLICT OF INTEREST STATEMENT

Actelion, a Johnson & Johnson company provided the funding for the study and the manuscript. Yuen Tsang and Sumeet Panjabi are employees of Janssen Scientific Affairs. Michael Stokes is an employee of Evidera, which received consulting fees for this study. Yong‐Jin Kim and Rong Tilney are former employees of Evidera.

## ETHICS STATEMENT

This study used data from Optum's de‐identified Clinformatics® Data Mart Database. The data are compliant with the Health Insurance Portability and Accountability Act. Therefore, institutional review board approval was not required.

## Supporting information


**Table S1.** Diagnostic codes used in this study.
**Table S2.** PAH‐specific Treatments.
**Table S3.** Composite Outcome of Disease Progression and Procedure Codes.

**Figure S1.** Study Timeline.
**Figure S2.** Main Drivers of Total Cost. **a)** All‐cause. **b)** PAH‐related.

## Data Availability

Data for this study may be obtained through a license with Optum, a US commercial database provider. Therefore, the authors cannot provide the raw data used in the analysis. More information on accessing the Clinformatics Data Mart Database can be obtained online (https://www.optum.com/business/life-sciences/real-world-data/claims-data.html).
