# Supplementary material for: Impact of selexipag use within 12 months of pulmonary arterial hypertension diagnosis on hospitalizations and medical costs: A retrospective cohort study
Source: Clin Respir J. 2023 Oct 7;17(12):1209–22. doi: 10.1111/crj.13704 (PMC10730465; doi:10.1111/crj.13704)
Supplement: Supplementary file 1 — Table S1. Diagnostic codes used in this study. Table S2. PAH‐specific Treatments. Table S3. Composite Outcome of Disease Progression and Procedure Codes. [file CRJ-17-1209-s002.docx]

# Supplementary Material

Table S1. Diagnostic codes used in this study

| Diagnosis Code | Code Description |
| --- | --- |
| ICD-9: 416.0, 416.8  ICD-10: I27.0, I27.20, I27.21, I27.89 | Pulmonary hypertension (PH) |
| ICD-9: 428.0  ICD-10: I50.81 | Right heart failure |

Table S2. PAH-specific Treatments

| Class | Generic name |
| --- | --- |
| Prostacyclin pathway agents (PPA)† | Selexipag  Epoprostenol  Iloprost  Treprostinil |
| Endothelin receptor antagonists (ERA)† | Bosentan  Ambrisentan  Macitentan |
| Soluble guanylate cycles stimulators (sGC) | Riociguat |
| Phosphodiesterase-5 inhibitors (PDE-5I)‡ | Sildenafil  Tadalafil |

†All available formulations were used

‡To ensure PDE-5I therapy was used to treat PAH, we focused only on appropriate branded agents and generic preparations with a dispending rate >15 pills per month^29^

Table S3. Composite Outcome of Disease Progression and Procedure Codes

| Condition | Definition |
| --- | --- |
| Receipt of parenteral PPA | At least one prescription dispense or outpatient office visit resulting in a relevant code for parenteral PPA |
| Lung transplantation | One or more inpatient claims with relevant procedure codes:  ICD-9-Procedure: 33.5, 33.50, 33.51, 33.52, 33.6  ICD-10-Procedure: 0BYK0Z0, 0BYL0Z0, 0BYK0Z1, 0BYL0Z1, 0BYK0Z2, 0BYL0Z2, 0BYM0Z0, 0BYM0Z0, 0BYM0Z1, 0BYM0Z1, 0BYM0Z2, 0BYM0Z2, 02YA0Z0, 02YA0Z1, 02YA0Z2  CPT codes: 32851, 32852, 32853, 32854, 33935 |
| Balloon atrial septostomy | One or more inpatient claims with relevant procedure codes:  ICD-9-Procedure: 35.4, 35.41, 35.42  ICD-10-Procedure: 02QA4ZZ, 02QA3ZZ, 02QA0ZZ, 02B50ZZ, 02B53ZZ, 02B54ZZ  CPT codes: 33737, 33735, 33736, 92992, 92993 |
