# Supplementary material for: Impact of selexipag use within 12 months of pulmonary arterial hypertension diagnosis on hospitalizations and medical costs: A retrospective cohort study
Source: Clin Respir J. 2023 Oct 7;17(12):1209–22. doi: 10.1111/crj.13704 (PMC10730465; doi:10.1111/crj.13704)
Supplement: Supplementary file 2 — Figure S1. Study Timeline. Figure S2. Main Drivers of Total Cost. a) All‐cause. b) PAH‐related. [file CRJ-17-1209-s001.pptx]

## Slide 1
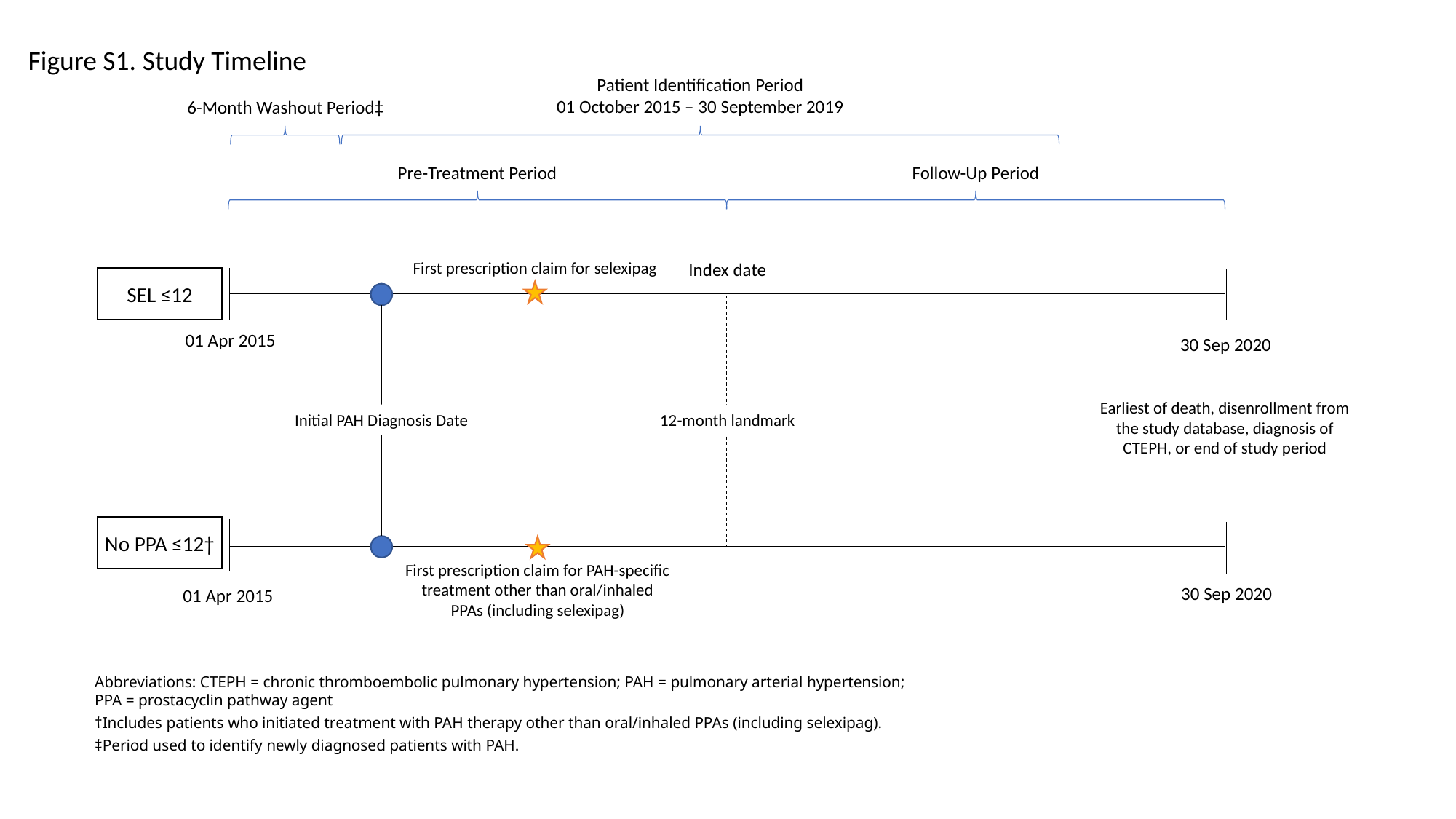

Figure S1. Study Timeline
Patient Identification Period
01 October 2015 – 30 September 2019
6-Month Washout Period‡
Pre-Treatment Period
Follow-Up Period
First prescription claim for selexipag
Index date
SEL ≤12
01 Apr 2015
30 Sep 2020
Earliest of death, disenrollment from the study database, diagnosis of CTEPH, or end of study period
Initial PAH Diagnosis Date
12-month landmark
No PPA ≤12†
First prescription claim for PAH-specific treatment other than oral/inhaled PPAs (including selexipag)
30 Sep 2020
01 Apr 2015
Abbreviations: CTEPH = chronic thromboembolic pulmonary hypertension; PAH = pulmonary arterial hypertension;
PPA = prostacyclin pathway agent
†Includes patients who initiated treatment with PAH therapy other than oral/inhaled PPAs (including selexipag).
‡Period used to identify newly diagnosed patients with PAH.

## Slide 2
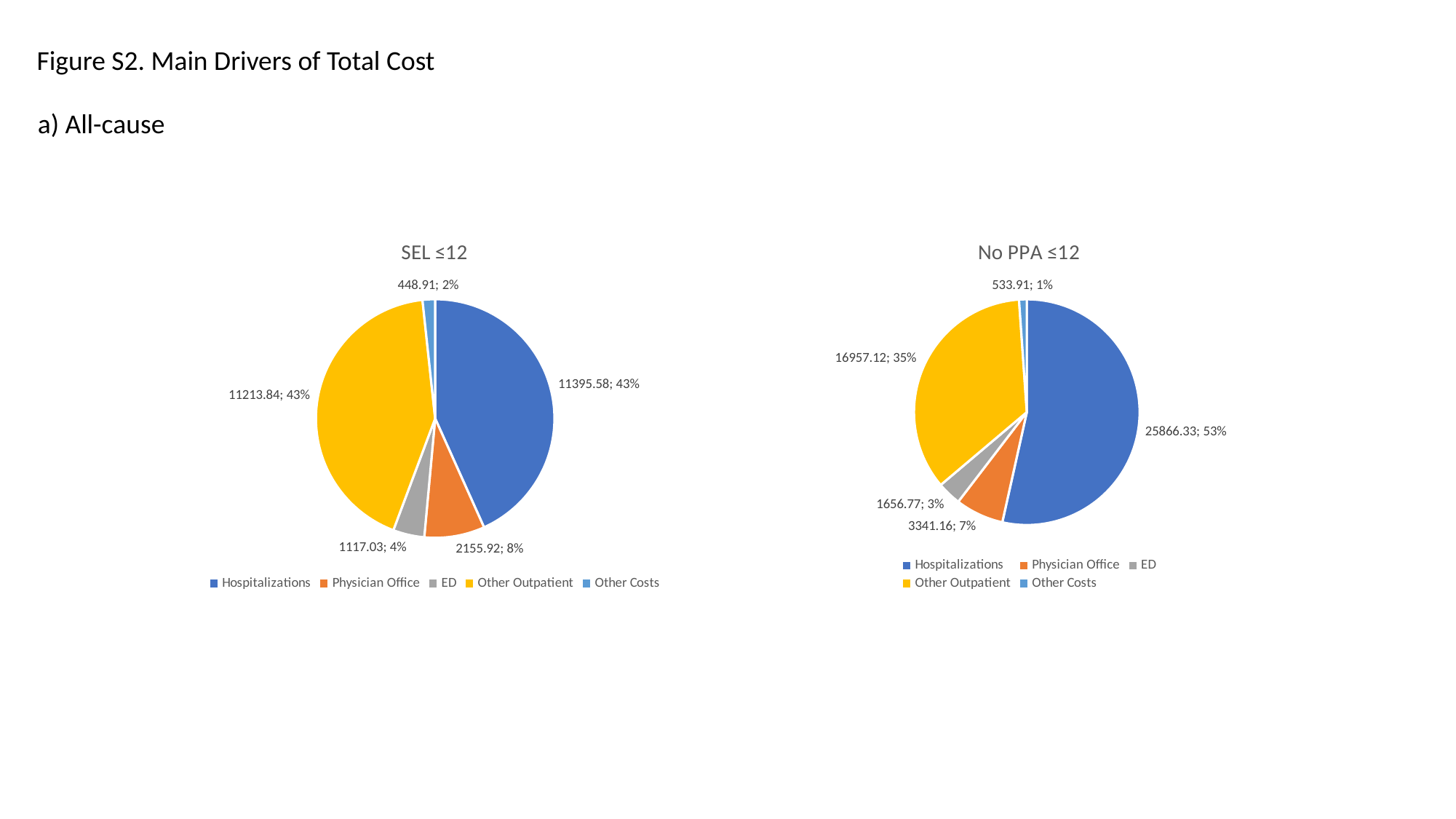

Figure S2. Main Drivers of Total Cost
a) All-cause
### Chart:
| Category | No PPA ≤12 |
|---|---|
| Hospitalizations | 25866.33 |
| Physician Office | 3341.16 |
| ED | 1656.77 |
| Other Outpatient | 16957.12 |
| Other Costs | 533.91 |
### Chart:
| Category | SEL ≤12 |
|---|---|
| Hospitalizations | 11395.58 |
| Physician Office | 2155.92 |
| ED | 1117.03 |
| Other Outpatient | 11213.84 |
| Other Costs | 448.91 |

## Slide 3
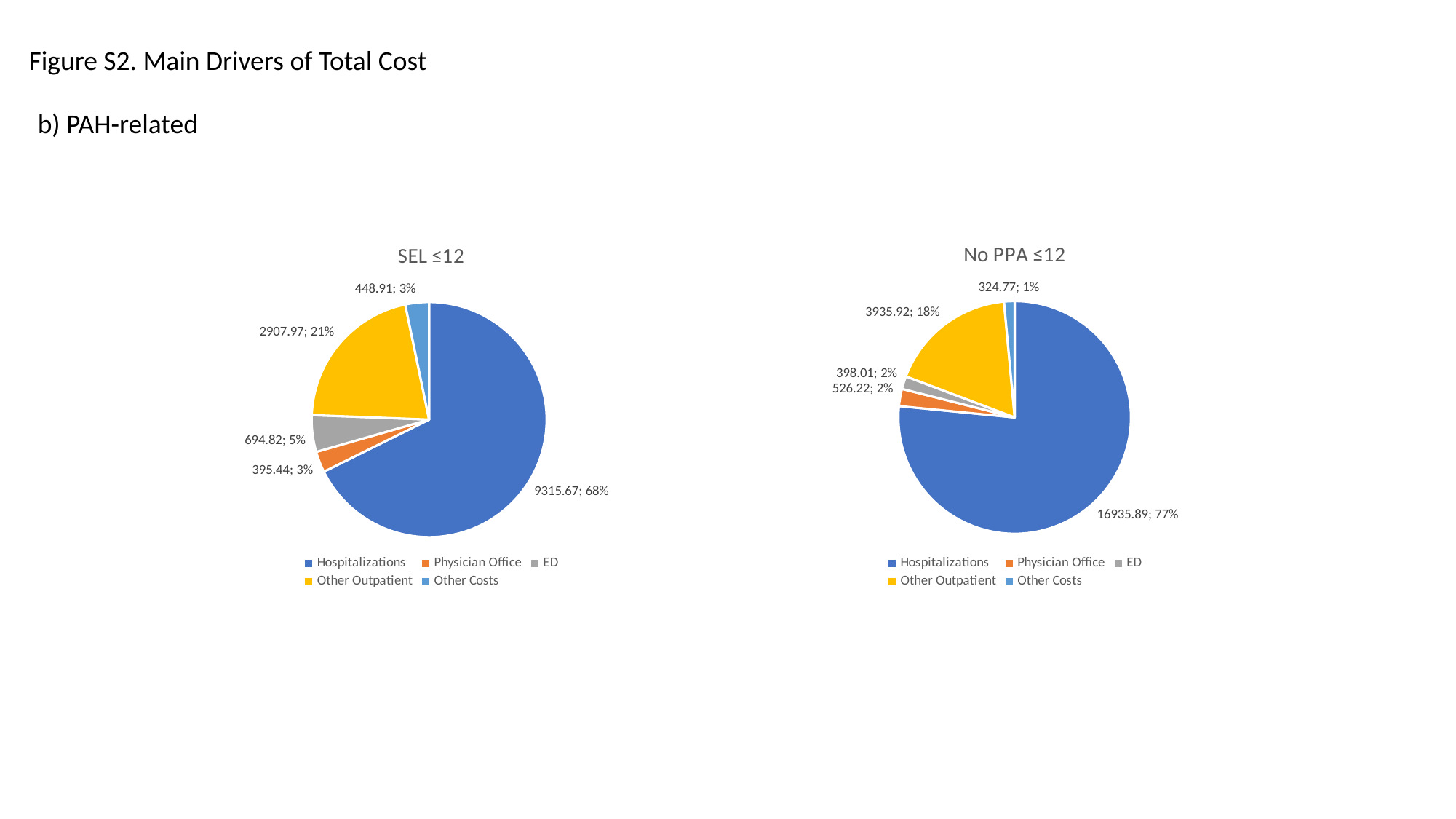

Figure S2. Main Drivers of Total Cost
b) PAH-related
### Chart:
| Category | No PPA ≤12 |
|---|---|
| Hospitalizations | 16935.89 |
| Physician Office | 526.22 |
| ED | 398.01 |
| Other Outpatient | 3935.92 |
| Other Costs | 324.77 |
### Chart:
| Category | SEL ≤12 |
|---|---|
| Hospitalizations | 9315.67 |
| Physician Office | 395.44 |
| ED | 694.82 |
| Other Outpatient | 2907.97 |
| Other Costs | 448.91 |
